# Access to choice‑enabled healthcare providers among children and adults with intellectual disabilities in comparison with the general population (IDcare)

**DOI:** 10.1186/s12913-026-14955-8

**Published:** 2026-06-18

**Authors:** Anna Axmon, Karin Engström, Magnus Sandberg

**Affiliations:** 1 Epidemiology and Bioinformatics, Division of Occupational and Environmental Medicine, Department of Laboratory Medicine, Lund University, Lund, Sweden; 2 Department of Health Sciences, Lund University, Lund, Sweden

**Keywords:** Patient involvement, Private healthcare providers, Intellectual disability, Register study, Primary healthcare, Specialist care, Health services accessibility

## Abstract

**Background:**

People with intellectual disabilities often have complex healthcare needs and may therefore experience different patterns of healthcare utilization. This study examined the overlap between the use of public and private healthcare providers as an indicator of access to choice-enabled healthcare providers within a patient-choice-based health system.

**Methods:**

We assessed overlap in two complementary ways: among people with at least one private healthcare contact and among those with at least one public healthcare contact in a cohort of people with intellectual disability (ID cohort) and a referent cohort from the general population (gPop cohort). The ID cohort comprised 2 813 children and 9 079 adults, whereas the gPop cohort comprised 146 048 children and 982 186 adults. Data regarding healthcare contacts in public and privately organized healthcare were collected from the Skåne Healthcare Register.

**Results:**

Among those with at least one contact in privately organized healthcare, both children and adults with intellectual disabilities were as likely as those in the general population to also have contacts in public primary healthcare and public somatic specialist care. However, they were more likely to have contacts in public psychiatric specialist care. Among those with at least one contact in public healthcare, children with intellectual disabilities were as likely as their age peers in the general population to also have contacts in privately organized healthcare. However, adults with intellectual disabilities were less likely than their age peers to have contacts in privately organized healthcare.

**Conclusions:**

These findings have important implications for patient‑choice‑based health systems. They indicate that formally available provider choice does not result in similar patterns of utilisation across populations. Also, that adults with intellectual disabilities may face challenges in navigating and using privately organised providers within such systems. The prominent role of public psychiatric specialist services further highlights the continued importance of publicly provided specialist care for this group. Together, the results underscore the need for patient‑choice‑based healthcare systems to be designed and implemented in ways that support accessibility, coordination, and navigation for people with intellectual disabilities, particularly adults with complex healthcare needs and during periods of healthcare disruption.

**Supplementary Information:**

The online version contains supplementary material available at 10.1186/s12913-026-14955-8.

## Background

In healthcare systems, one policy approach intended to strengthen patient’s influence over their healthcare is the introduction of patient choice systems, in which people are free to choose between different healthcare providers. During the early 2000s, several European countries introduced such systems, allowing people to choose between public and private healthcare providers within a patient-choice-based health system, with the aim of increasing patients’ influence over their care and improving healthcare quality through competition [1–3]. These reforms can be conceptually linked to the broader shift towards person-centred care, which emphasises responsiveness to patients’ needs and preferences [4, 5]. In healthcare systems that formally allow patients to choose between public and private healthcare providers, the use of more than one type of provider can be viewed as an indicator of realised use of the available choice options within the system. However, evidence of consistent positive effects is limited, including for waiting times [6–8], number of visits [6], mortality [8–10], hospital length of stay [8–10], and hospital readmissions [8–10]. Instead, the introduction of free choice of care has been associated with increased inequities [11–14] and may also complicate the delivery of integrated care [15, 16].

Intellectual disability is a condition that is present from early life and diagnosed before the age of 18 years, characterized by limited cognitive ability and understanding. People with intellectual disabilities are known to have poorer somatic [17] and mental [18] health than the general population, and they often have complex healthcare needs. As a result, people with intellectual disabilities may be particularly vulnerable in healthcare systems that rely on patients’ ability to navigate available options and actively choose between providers. This concern is supported by studies showing that people with intellectual disabilities experience barriers in shared decision-making [19–21], despite expressing a desire to be involved in decisions regarding their own healthcare [22, 23] and evidence that supported decision-making can be successfully implemented in some contexts [24–26], particularly when appropriate support is provided [22, 27, 28], and research showing that self-determination is associated with mental health [29, 30], quality of life [31], and life satisfaction [32]. Little is known about how people with intellectual disabilities utilise patient choice options within healthcare systems that formally offer free choice of provider.

In studies of healthcare choice, it is important to consider that for children – both with and without disabilities – seeking healthcare relies more on the parent than on the child. Therefore, there may be different processes in the choice of healthcare providers for children and adults. Such differences can be identified only by assessing children and adults separately.

The COVID-19 pandemic had a major impact on healthcare services. In Sweden, the high demand put on the healthcare system resulted in, e.g., a decrease in surgeries and somatic inpatient care and fewer contacts with physicians in specialist outpatient care [33]. Although previous studies have not found clear evidence of discrimination against children [34] or older people [35] with intellectual disabilities, studies on healthcare utilization need to consider differences in healthcare delivery between the pre-pandemic and pandemic periods.

## Methods

This register-based study uses national and regional registers to both identify the study population and collect outcome data. We operationalised access to choice-enabled providers as observed utilisation across provider types, which reflects realised use of available options rather than the availability of choice per se or people’ ability to actively choose providers. Thus, the aim of the present study was to assess the overlap between the use of public and private healthcare providers as an indicator of access to choice-enabled healthcare providers within a patient-choice-based health system among children and adults with intellectual disabilities in comparison to the general population during pre-pandemic (2014–2019) and pandemic periods (2020–2021).

### Setting

This study is set in Skåne (Scania), the southernmost region of Sweden. In September of 2025, the population of Skåne was approximately 1.4 million, which corresponds to approximately 13% of the Swedish population. The administrative unit for Skåne, i.e., the county council, is called Region Skåne.

Swedish healthcare is part of the welfare system and is financed mainly by taxes. Patients pay only a small fee, which may vary across counties. In Skåne, the patient fee for consultations with physicians in primary healthcare and outpatient specialist care is 200 SEK (approximately €17, conversion made November 24, 2025) [36]. In all of Sweden, there is a yearly cap of 1450 SEK [37], corresponding to approximately €122. Thus, a patient would never pay more than this amount over a 12-month period. Basic healthcare is provided in primary healthcare. These may be either public or privately organized. Healthcare requiring specialized care is provided at hospitals or by private healthcare providers. The fee is the same in public healthcare and in privately organized healthcare, provided that the private healthcare provider is under contract with the county council.

According to the Swedish Act on System of Choice in the Public Sector [38], which was introduced in 2009, patients are free to choose their own healthcare provider in both primary and specialist care. This legislation establishes a formal patient-choice-based healthcare system, in which patients may choose between publicly and privately operated providers included in the regional healthcare system. Consequently, privately organized healthcare within the regional system represents access to alternative, choice-enabled healthcare providers rather than privately financed care.

The availability of private healthcare providers varies between types of care. For example, approximately 50% of all physical contacts in primary healthcare are to private healthcare providers, but most of these are not to physicians but to physiotherapists and occupational therapists [39]. In both somatic and psychiatric specialist care, approximately 20% of healthcare contacts are to private healthcare providers, the majority of which are in an open care setting. However, most hospitals are owned and operated by regions [40]. Approximately 86% of all expenditures on healthcare are public expenditures [41], suggesting that healthcare contacts with private healthcare providers not under contract with county councils are limited.

According to the Swedish Act Concerning Support and Service for Persons with Certain Functional Impairments (the LSS law), people with intellectual disability and/or autism spectrum disorder are entitled to the service and support they need to live fulfilling lives. These services and support include, e.g., daily activities, housing, and personal assistance but not financial aid. The services and support are provided by the municipality after application from the individual or their care takers, followed by a need assessment. As a rule, support according to the LSS act is free of charge, but the municipality may charge for costs associated with supported living (mainly rent) and for leisure time and cultural activities.

### Data sources

The Skåne Healthcare Register comprises data on all public healthcare and healthcare provided by private healthcare providers under contract with Region Skåne. Each record includes the date of the healthcare contact, which healthcare area (e.g., primary healthcare or somatic specialist care) the contact was recorded in, the profession of the treating person, and the type of contact (e.g., in person or phone) as well as information on the patient, such as sex and age. Moreover, records from public healthcare comprise both primary and secondary diagnoses according to the International Statistical Classification of Diseases and Related Health Problems 10th Revision (ICD-10).

The LSS register at the Swedish National Board of Health and Welfare includes data on all services and support provided according to the LSS law. Although a diagnosis of intellectual disability or autism spectrum disorder is required to be eligible for LSS support, diagnoses are not recorded in the LSS register.

### Study populations

This study is based on the IDcare study [42]. In IDcare, we included all people living in Skåne on January 1st, 2014. People with at least one measure of service and support according to the LSS act from 2007 to 2021 and/or people with at least one diagnosis of intellectual disability (F70–F79 in the ICD-10) or Down syndrome (Q90) from 2014 to 2021 were included in the ID cohort (*n* = 14 716; 5 939 women and 8 777 men). As people living with a person with intellectual disabilities may differ from people not living with such a person regarding health and healthcare utilization, we excluded those who were not in the ID cohort but in the same family or household as a person in the ID cohort (*n* = 31 688). The remaining population of Skåne comprised the general population reference cohort (gPop cohort, *n* = 1 226 955; 620 032 women and 606 923 men). For the present study, we identified those who were either **children** (i.e., < 18 years old, corresponding to being born from 2004 to 2014) or **adults** (i.e., > 18 years old, corresponding to being born before 1996) during the entire study period, excluding those aged 18 years from 2014 to 2021. If not otherwise implied, the terms men and women will henceforth be used to describe the adult population by sex and boys and girls to describe the children by sex.

### Healthcare utilization

Data regarding healthcare utilization were collected from the Skåne Healthcare Register. We selected all physical contacts (i.e., excluding phone and video consultations) with physicians if they were recorded in healthcare areas public primary healthcare (henceforth referred to as **primary healthcare**), privately organized healthcare for care providers under contract with Region Skåne (**private healthcare**), public psychiatric outpatient specialist care (**psychiatric healthcare**), or public somatic outpatient specialist care (**somatic healthcare**). For each healthcare area, we determined whether each person had at least one visit during the pre-pandemic (2014–2019) and pandemic (2020–2021) periods.

### Statistics

We assessed the overlap between public and private healthcare utilization within the publicly funded healthcare system in two different ways. (1) Among people with at least one visit in **private** healthcare during each period (pre-pandemic and pandemic), we compared the number of people with at least one visit during the study period in each of the public healthcare areas in the ID cohort with that in the gPop cohort. (2) Among people with at least one visit in each **public** healthcare area during each period, we assessed the number of people with at least one visit during the study period in private healthcare.

Comparisons of the ID cohort to the gPop cohort were performed using Poisson regression to estimate relative risks (RRs) with 95% confidence intervals (CIs). All analyses were stratified by age group (children and adults) and sex, and adjusted for year of birth. The interaction between age group and cohort, i.e., whether the potential association between the ID cohort and the outcome differed between children and adults, was assessed via models including age group, cohort, and the cross-term between the age group and cohort. Similarly, the interaction between sex and cohort was assessed in models including sex, cohort, and the cross-term between sex and cohort, and the interaction between period and cohort was assessed in models including period, cohort, and the cross-term between period and cohort.

All analyses were performed in IBM SPSS Statistics 30.0. p values less than 0.05 were considered statistically significant. To avoid the identification of single people as well as in consideration of statistical power, results are presented only for groups comprising at least five people.

## Results

### Population characteristics

The ID cohort comprised 2 813 children (1 864 boys and 949 girls) and 9 079 adults (5 154 men and 3 925 women), whereas the gPop cohort comprised 146 048 children (74 526 boys and 71 522 girls) and 982 186 adults (481 843 men and 500 343 women). The children in the ID cohort were slightly older than the children in the gPop cohort were (median year of birth 2008 vs. 2009), whereas the opposite was observed among the adults (median year of birth 1981 vs. 1965).

### Healthcare utilization

The percentage of people with at least one contact in **private healthcare** ranged from 5% among boys and girls in the ID cohort during the pandemic period to 70% among women in the gPop cohort during the pre-pandemic period (Table 1). Among adults, there was a pattern of greater use among women than among men in both cohorts and during both periods. The percentage of people with at least one contact in **any public healthcare** was high in both cohorts, with almost 100% among children and adults in the ID cohort during the pre-pandemic period.

**Table 1:** Number and percentage of people with at least one contact in each type of healthcare

Period	Healthcare area	Sex	Children				Adults			
			gPop		ID		gPop		ID	
			n	%	n	%	n	%	n	%
Pre-pandemic	Private healthcare	Boys/men	15 205	20	394	21	149 446	31	1 023	20
		Girls/women	13 162	18	190	20	348 195	70	1 935	49
	Any public healthcare	Boys/men	69 930	94	1 835	98	431 311	90	5 030	98
		Girls/women	66 633	93	939	99	473 009	95	3 852	98
	Primary healthcare	Boys/men	66 516	89	1 668	89	406 190	84	4 807	93
		Girls/women	63 708	89	857	90	456 978	91	3 766	96
	Psychiatric healthcare	Boys/men	4 530	6	870	47	31 528	7	2 190	42
		Girls/women	2 594	4	373	39	34 768	7	1 614	41
	Somatic healthcare	Boys/men	54 630	73	1 645	88	346 628	72	3 813	74
		Girls/women	49 715	70	871	92	406 578	81	3 273	83
Pandemic	Private healthcare	Boys/men	4 565	6	93	5	56 953	13	263	5
		Girls/women	4 467	6	46	5	242 028	52	1 096	30
	Any public healthcare	Boys/men	47 566	64	1 489	80	313 686	70	4 112	86
		Girls/women	45 698	64	825	88	369 081	79	3 248	89
	Primary healthcare	Boys/men	36 915	50	935	50	271 035	60	3 558	74
		Girls/women	37 085	52	567	60	331 008	71	2 935	81
	Psychiatric healthcare	Boys/men	4 573	6	444	24	15 567	3	1 292	27
		Girls/women	3 978	6	221	23	17 028	4	970	27
	Somatic healthcare	Boys/men	28 040	38	1 071	58	213 776	48	2 513	52
		Girls/women	25 884	36	629	67	263 436	57	2 235	61

ID = a cohort of people with intellectual disabilities, gPop = a referent cohort of people from the general population

### Any public healthcare vs. private healthcare

Among those with at least one contact in private healthcare, men and women in the ID cohort were 17% and 11%, respectively, more likely than those in the gPop cohort to have at least one contact in public healthcare during the pandemic period (Table 2; Supplement 1). Although the risk estimates were similar for adults and children, the risks for boys and girls did not achieve statistical significance. Moreover, there were no statistically significant sex, age, or period effects.

Among those with at least one contact in public healthcare, both men and women in the ID cohort were less likely to have contact in private healthcare during both periods. The effect was more pronounced among men and during the pandemic period. For children, no difference in the risk of private healthcare was found between the cohorts for the pre-pandemic period, but during the pandemic period, both boys and girls in the ID cohort had a decreased risk of having at least one contact in private healthcare.

**Table 2:** Any public healthcare among people with intellectual disabilities compared with the general population

			*Children*					*Adults*					
			*gPop*		*ID*		*ID vs. gPop*	*gPop*		*ID*		*ID vs. gPop*	
Outcome	Period	Sex	n	%	n	%	RR (95% CI)	n	%	n	%	RR (95% CI)	p (age)
Any public healthcare among those with private healthcare	Pre-pandemic	Boys/men	14 886	98	391	99	1.02 (0.92–1.12)	145 375	97	1 013	99	1.03 (0.97–1.10)	0.742
		Girls/women	12 862	98	189	99	1.02 (0.88–1.18)	339 425	97	1 913	99	1.02 (0.97–1.07)	0.987
		p (sex)					0.965					0.838	
	Pandemic	Boys/men	3 536	77	85	91	1.17 (0.95–1.46)	50 216	88	252	96	**1.17** (**1.04**–**1.33**)	0.989
		Girls/women	3 573	80	44	96	1.17 (0.87–1.58)	209 878	87	1 029	94	**1.11** (**1.05**–**1.18**)	0.665
		p (sex)					0.976					0.580	
	p (period)	Boys/men					0.208					0.356	
		Girls/women					0.341					0.110	
Private healthcare among those with any public healthcare	Pre-pandemic	Boys/men	14 886	21	391	21	1.02 (0.92–1.13)	145 375	34	1 013	20	**0.69** (**0.65**–**0.73**)	**< 0.001**
		Girls/women	12 862	19	189	20	1.04 (0.90–1.21)	339 425	72	1 913	50	**0.71** (**0.68**–**0.74**)	**< 0.001**
		p (sex)					0.619					**< 0.001**	
	Pandemic	Boys/men	3 536	7	85	6	**0.77** (**0.62**–**0.96**)	50 216	16	252	6	**0.47** (**0.41**–**0.53**)	**< 0.001**
		Girls/women	3 573	8	44	5	**0.68** (**0.50**–**0.91**)	209 878	57	1 029	32	**0.55** (**0.52**–**0.59**)	0.166
		p (sex)					0.525					**< 0.001**	
	p (period)	Boys/men					**0.026**					**< 0.001**	
		Girls/women					**0.012**					**< 0.001**	

Relative risks (RRs) with 95% confidence intervals (CIs) for **any public healthcare** among children and adults with intellectual disabilities compared with their age peers in the general population, with p values assessing risk differences between sexes [p (sex)], age groups [p (age)], and periods [p (period)]. Bold text indicates statistically significant results

### Primary healthcare vs. private healthcare

Among those with at least one contact in private healthcare, there was a 26% increased risk for men and a 19% increased risk for women in the ID cohort to have at least one contact in primary healthcare during the pandemic period (Table 3, Supplement 1). For women, this risk was statistically significantly greater than that in the pre-pandemic period. No effects were found among boys and girls in the ID cohort, nor were there any other statistically significant age, sex, or period effects.

Among those with at least one contact in primary healthcare, adults had a decreased risk of having at least one contact in private healthcare. The risk associated with the ID cohort was more pronounced among men and during the pandemic period. No effects were found for the children in the ID cohort.

**Table 3:** Primary healthcare among people with intellectual disabilities compared with the general population

			*Children*					*Adults*					
			*gPop*		*ID*		*ID vs. gPop*	*gPop*		*ID*		*ID vs. gPop*	p (age)
Outcome	Period	Sex	n	%	n	%	RR (95% CI)	n	%	n	%	RR (95% CI)	
Public primary healthcare among those with private healthcare	Pre-pandemic	Boys/men	14 379	95	363	92	0.98 (0.88–1.09)	139 298	93	980	96	1.05 (0.99–1.12)	0.213
		Girls/women	12 438	94	182	96	1.02 (0.88–1.18)	330 255	95	1 886	97	1.03 (0.99–1.08)	0.794
		p (sex)					0.671					0.874	
	Pandemic	Boys/men	2 820	62	57	61	0.99 (0.76–1.28)	44 771	79	233	89	**1.26** (**1.11**–**1.44**)	0.099
		Girls/women	2 936	66	33	72	1.07 (0.76–1.50)	188 777	78	975	89	**1.19** (**1.12**–**1.27**)	0.603
		p (sex)					0.697					0.581	
	p (period)	Boys/men					0.886					0.209	
		Girls/women					0.683					**0.012**	
Private healthcare among those with public primary healthcare	Pre-pandemic	Boys/men	14 379	22	363	22	1.02 (0.92–1.13)	139 298	34	980	20	**0.68** (**0.64**–**0.72**)	**< 0.001**
		Girls/women	12 438	20	182	21	1.09 (0.94–1.26)	330 255	72	1 886	50	**0.71** (**0.68**–**0.74**)	**< 0.001**
		p (sex)					0.376					**< 0.001**	
	Pandemic	Boys/men	2 820	8	57	6	0.80 (0.62–1.04)	44 771	17	233	7	**0.47** (**0.42**–**0.54**)	**0.001**
		Girls/women	2 936	8	33	6	0.73 (0.52–1.02)	188 777	57	975	33	**0.57** (**0.53**–**0.60**)	0.143
		p (sex)					0.706					**< 0.001**	
	p (period)	Boys/men					0.107					**< 0.001**	
		Girls/women					**0.040**					**< 0.001**	

Relative risks (RRs) with 95% confidence intervals (CIs) for **primary healthcare** among children and adults with intellectual disabilities compared with their age peers in the general population, with p values assessing risk differences between sexes [p (sex)], age groups [p (age)], and periods [p (period)]. Bold text indicates statistically significant results

### Psychiatric healthcare vs. private healthcare

Among those with at least one contact in private healthcare, children and adults in the ID cohort were up to nine times more likely to have at least one contact in psychiatric healthcare (Table 4, Supplement 1). During the pre-pandemic period, the risk was greater among children than among adults, whereas the opposite was found during the pandemic period. Moreover, whereas the risk decreased over time for children, it increased for adults.

Among those with at least one contact in psychiatric healthcare, adults had a decreased risk of having at least one contact in private healthcare. The effect was more pronounced during the pandemic period and among men. Among children, particularly girls, there was a decreased risk of having at least one contact in private healthcare during the pandemic but not during the pre-pandemic period.

**Table 4:** Psychiatric healthcare among people with intellectual disabilities compared with the general population

			*Children*					*Adults*					
			*gPop*		*ID*		*ID vs. gPop*	*gPop*		*ID*		*ID vs. gPop*	p (age)
Outcome	Period	Sex	n	%	n	%	RR (95% CI)	n	%	n	%	RR (95% CI)	
Public psychiatric healthcare among those with private healthcare	Pre-pandemic	Boys/men	989	7	206	52	**7.74** (**6.66**-**9.00**)	11 210	8	557	54	**5.01** (**4.60**–**5.47**)	**< 0.001**
		Girls/women	589	4	83	44	**9.31** (**7.40**-**11.71**)	24 245	7	898	46	**5.25** (**4.91**–**5.62**)	**< 0.001**
		p (sex)					0.139					0.257	
	Pandemic	Boys/men	309	7	29	31	**4.35** (**2.97**–**6.37**)	2 049	4	88	33	**5.78** (**4.65**–**7.17**)	0.335
		Girls/women	345	8	10	22	**2.24** (**1.19**–**4.20**)	8 004	3	321	29	**7.10** (**6.35**–**7.95**)	**0.006**
		p (sex)					0.121					0.087	
	p (period)	Boys/men					**0.005**					**0.037**	
		Girls/women					**< 0.001**					**< 0.001**	
Private healthcare among those with public psychiatric healthcare	Pre-pandemic	Boys/men	989	22	206	24	1.03 (0.89–1.20)	11 210	36	557	25	**0.78** (**0.72**–**0.85**)	**< 0.001**
		Girls/women	589	23	83	22	0.95 (0.75–1.20)	24 245	70	898	56	**0.84** (**0.78**–**0.89**)	0.178
		p (sex)					0.438					0.061	
	Pandemic	Boys/men	309	7	29	7	0.97 (0.66–1.42)	2 049	13	88	7	**0.60** (**0.49**–**0.75**)	**0.036**
		Girls/women	345	9	10	5	**0.52** (**0.28**–**0.97**)	8 004	47	321	33	**0.77** (**0.69**–**0.86**)	0.234
		p (sex)					0.095					**0.014**	
	p (period)	Boys/men					0.767					**0.004**	
		Girls/women					0.071					**0.046**	

Relative risks (RRs) with 95% confidence intervals (CIs) for **psychiatric healthcare** among children and adults with intellectual disabilities compared with their age peers in the general population, with p values assessing risk differences between sexes [p (sex)], age groups [p (age)], and periods [p (period)]. Bold text indicates statistically significant results

### Somatic healthcare vs. private healthcare

Among those with at least one contact in private healthcare, there was a pattern of increased risk of having at least one contact in somatic healthcare (Table 5; Supplement 1). Although not always statistically significant, this pattern was consistent across age groups, sexes, and periods.

Among those with at least one contact in somatic healthcare, adults with ID were less likely than those in the gPop cohort were to have at least one contact in private healthcare. The effect was more pronounced among men than among women and during the pandemic period. Among children, a decreased risk of having at least one contact in private healthcare was found only during the pandemic period, with a greater effect among girls than among boys.

**Table 5:** Somatic healthcare among people with intellectual disabilities compared with the general population

			*Children*					*Adults*					
			*gPop*		*ID*		*ID vs. gPop*	*gPop*		*ID*		*ID vs. gPop*	p (age)
Outcome	Period	Sex	n	%	n	%	RR (95% CI)	n	%	n	%	RR (95% CI)	
Public somatic healthcare among those with private healthcare	Pre-pandemic	Boys/men	12 619	83	364	92	**1.12** (**1.01**–**1.24**)	127 561	85	826	81	1.04 (0.97–1.11)	0.291
		Girls/women	10 682	81	180	95	**1.17** (**1.01**–**1.36**)	296 546	85	1 676	87	**1.05** (**1.00**-**1.10**)	0.187
		p (sex)					0.614					0.290	
	Pandemic	Boys/men	2 284	50	62	67	**1.32** (**1.03**–**1.71**)	38 453	68	202	77	**1.39** (**1.21**–**1.59**)	0.750
		Girls/women	2 293	51	30	65	1.24 (0.86–1.77)	150 567	62	739	67	**1.16** (**1.08**–**1.25**)	0.672
		p (sex)					0.798					0.089	
	p (period)	Boys/men					0.187					**0.020**	
		Girls/women					0.642					0.240	
Private healthcare among those with public somatic healthcare	Pre-pandemic	Boys/men	12 619	23	364	22	0.98 (0.88–1.08)	127 561	37	826	22	**0.66** (**0.62**–**0.71**)	**< 0.001**
		Girls/women	10 682	21	180	21	0.96 (0.83–1.12)	296 546	73	1 676	51	**0.71** (**0.68**–**0.75**)	**< 0.001**
		p (sex)					0.930					**< 0.001**	
	Pandemic	Boys/men	2 284	8	62	6	**0.71** (**0.55**–**0.92**)	38 453	18	202	8	**0.54** (**0.47**–**0.62**)	0.059
		Girls/women	2 293	9	30	5	**0.53** (**0.37**–**0.77**)	150 567	57	739	33	**0.57** (**0.53**–**0.61**)	0.788
		p (sex)					0.214					**0.001**	
	p (period)	Boys/men					**0.028**					**0.001**	
		Girls/women					**0.003**					**< 0.001**	

Relative risks (RRs) with 95% confidence intervals (CIs) for **somatic healthcare** among children and adults with intellectual disabilities compared with their age peers in the general population, with p values used to assess risk differences between sexes, age groups, and periods. Bold text indicates statistically significant results

## Discussion

In this register‑based study, we examined overlap between the use of public and privately organised healthcare providers among children and adults with intellectual disabilities and compared these patterns with those observed in the general population. Among adults with intellectual disabilities who had contacts in public primary or specialist care, the likelihood of also having contacts with privately organized healthcare providers was lower than among adults in the general population, whereas no such difference was observed among children. In contrast, among people with contacts in privately organized healthcare, both children and adults with intellectual disabilities had similar use of public primary and somatic specialist care compared with the general population, but a higher use of public psychiatric outpatient specialist care. These patterns were observed across sexes and during both the pre‑pandemic and pandemic periods, with some variation by age group, sex, and period.

Before interpreting the broader implications of these findings, it is important to consider the strengths and limitations of the present study. A major strength is the use of population-based registers to identify both the study cohorts and healthcare utilization outcomes. The registers are maintained by national and regional authorities and cover all residents in the region, resulting in minimal risk of selection bias. A further strength is the inclusion of all people in a defined geographical area comprising both urban and rural settings and representing more than a tenth of the Swedish population, which supports the generalisability of the findings to larger populations, particularly in other welfare-based healthcare systems. In addition, the long study period increased the likelihood of capturing even infrequent use of privately organized healthcare providers.

A potential weakness of the study is the limited information available on privately organized healthcare. The available data did not allow us to distinguish between different levels of care (e.g., primary vs. specialist care) provided by private healthcare providers. However, by relating privately organized healthcare to all types of public healthcare (primary healthcare, specialist care, and all public healthcare combined), we were able to assess overlap in healthcare utilization across different levels of healthcare. Moreover, as diagnoses from privately organized healthcare were not available from the Skåne Healthcare Register, we were unable to identify clinical characteristics associated with a greater likelihood of using public or private healthcare, respectively. Even so, the aim of the study was to describe patterns of overlap between public and privately organized healthcare utilization, and not to examine the underlying reasons for choosing one or the other. A related concern is that we did not include private healthcare providers that are not under contract with Region Skåne. However, although private health financing accounted for approximately 14% of current health expenditure in Sweden in 2020, the majority (93%) of this consisted of households’ out-of-pocket payments, i.e., patient fees within the publicly funded healthcare system rather than fully privately financed care [41]. This suggests that healthcare provided entirely outside the regional healthcare system likely constitutes a very small proportion of overall healthcare utilisation in Sweden. Thus, excluding private healthcare providers not under contract with Region Skåne should not pose a threat to the validity of this study.

Another potential limitation is the use of a binary measure of healthcare utilisation (having at least one contact during each period) rather than, for example, the number or proportion of contacts in privately organized healthcare. This approach was chosen to capture whether people utilised more than one type of healthcare provider within the system. Nevertheless, it limits conclusions regarding the intensity or frequency of healthcare utilization. In addition, the pre-pandemic and pandemic periods differed in length, which restricts direct comparisons of utilisation levels between periods. However, as the analyses focused on differences between cohorts within each period rather than changes over time within cohorts, this discrepancy is unlikely to have materially affected the results.

A final weakness is the potential misclassification of people with intellectual disability due to the limited time period used to identify the ID cohort. Such misclassification would have attenuated rather than exaggerated differences between the cohorts. However, given the large size of the gPop cohort, the overall impact on the findings is likely limited.

Only physical healthcare contacts were included while telephone and video contacts were not. This is likely to have had the greatest impact during the pandemic period, when the use of digital consultations increased. We have previously reported that during the pandemic, people with intellectual disabilities were more likely than the general population to have video contact with specialist healthcare but less likely to have such contact with primary healthcare physicians [43]. As the observed associations for the ID cohort were stronger during the pandemic period for both primary and specialist care, restricting the analyses to physical contacts is unlikely to have substantially biased the results.

We would like to highlight a few of our findings. First, among people with at least one contact in privately organized healthcare, the proportion who also had at least one contact in public healthcare was high and similar in the ID and gPop cohorts, across age groups and sexes. This indicates substantial overlap in provider use, with privately organized healthcare commonly occurring alongside public healthcare. Because our analyses were restricted to physical visits to physicians, these patterns reflect physician-delivered care within these settings. When stratifying by type of public healthcare, overlap was similarly high for public primary healthcare and public somatic outpatient specialist care, although children with intellectual disabilities who used privately organized healthcare had a slightly higher likelihood of also having somatic specialist contacts. This pattern may be consistent with higher healthcare needs and more complex care pathways among children with intellectual disabilities, where privately organized healthcare alone may not cover all required services. Previous studies have reported that choice reforms can make integrated care for people with complex needs more difficult [15, 16]. Future investigations should examine whether differences in overlap are explained by variations in need, service availability, or care pathways, and to what extent they reflect barriers to obtaining coordinated care.

Second, psychiatric outpatient specialist care showed a distinct pattern: among people with at least one contact in privately organized healthcare, children and adults with intellectual disabilities were markedly more likely than their peers in the general population to also have contacts in public psychiatric outpatient specialist care. In other words, psychiatric specialist contacts among people using privately organized healthcare were substantially more common in the public sector for the ID cohort. One possible explanation is that psychiatric specialist care for people with intellectual disabilities may more often be delivered through established public specialist services with long-term follow-up, which could reduce the extent of psychiatric healthcare delivered by privately organised providers. Further research is needed to clarify how service organisation and continuity of care contribute to this pattern.

Third, among people with at least one contact in public healthcare, children in the ID cohort had similar likelihood of also having contacts in privately organized healthcare as children in the general population, whereas adults with intellectual disabilities were consistently less likely than their peers to have contacts in privately organized healthcare. This indicates lower utilisation of privately organised healthcare providers among adults with intellectual disabilities within a system that formally allows provider choice. A plausible explanation for the difference between children and adults is that healthcare contacts for children are more often initiated and managed by parents or caregivers, whereas adults may need to navigate provider options more independently or with variable support. These findings may reflect previously described barriers to healthcare among people with intellectual disabilities, including difficulties navigating healthcare systems, communicating healthcare needs, and accessing appropriate support in decision-making. At the same time, the findings may also indicate that patient-choice-based healthcare systems place greater demands on patients’ ability to independently navigate available providers and care pathways. Thus, the organisation of healthcare services may itself contribute to differences in healthcare accessibility and utilisation. Future studies should explore which factors are associated with the use of privately organised providers among adults with intellectual disabilities, including health status, severity of disability, sociodemographic factors, and social support, and should also investigate the observed sex differences. From a health‑system perspective, support functions that facilitate navigation and coordination, such as care coordinators, may be relevant to reduce fragmentation and help ensure that provider choice is accessible for people with intellectual disabilities. Other potentially important measures may include strengthening communication competence in both public and privately organised services, providing accessible information regarding available healthcare providers and care pathways, facilitating supported decision-making, and expanding accessible digital services and e-health options. The findings may also indicate that patient-choice-based healthcare systems are not always designed with the needs of people with intellectual disabilities and complex healthcare needs in mind.

## Conclusions

The findings from the present study have important implications for patient‑choice‑based health systems. They indicate that formally available provider choice does not result in similar patterns of utilisation across populations. Also, that adults with intellectual disabilities may face challenges in navigating and using privately organised providers within such systems. The prominent role of public psychiatric specialist services further highlights the continued importance of publicly provided specialist care for people with intellectual disabilities. Together, the results underscore the need for patient‑choice‑based healthcare systems to be designed and implemented in ways that support accessibility, coordination, and navigation for people with intellectual disabilities, particularly adults with complex healthcare needs and during periods of healthcare disruption. 

## Supplementary Information

Below is the link to the electronic supplementary material.

Supplementary Material 1

## Data Availability

Since the data comprise information about single people and are detailed enough to enable identification of, at least, some of the people included, they cannot be made publicly available. However, as the database was compiled from national register data, other researchers may contact the register holders to obtain access to the registries used in this study and thereby generate an identical database.

## Abbreviations

CI: Confidence interval
ICD-10: International Statistical Classification of Diseases and Related Health Problems 10th Revision
RR: Relative risk
