# Supplementary material for: Access to choice‑enabled healthcare providers among children and adults with intellectual disabilities in comparison with the general population (IDcare)
Source: BMC Health Serv Res. 2026 Jun 18;26:850. doi: 10.1186/s12913-026-14955-8 (PMC13282862; doi:10.1186/s12913-026-14955-8)
Supplement: Supplementary file 1 — Supplementary Material 1 [file 12913_2026_14955_MOESM1_ESM.docx]

Supplement 1 Healthcare among people with intellectual disabilities compared with the general population. Relative risks (RRs) with 95% confidence intervals (CIs) among children and adults with intellectual disabilities compared with their age peers in the general population, with p values assessing risk differences between sexes [p (sex)], age groups [p (age)], and periods [p (per)].

|  | | | | | **Children** | | | | **Adults** | | | | **p (age)** |
| --- | --- | --- | --- | --- | --- | --- | --- | --- | --- | --- | --- | --- | --- |
|  |  |  |  |  | **RR** | **95% CI** | |  | **RR** | **95% CI** | |  |  |
| Any public health-care | Public healthcare among those with private healthcare | Pre-pandemic | Boys/ men | Crude | 1.01 | 0.92 | 1.12 |  | 1.02 | 0.96 | 1.08 |  | 0.944 |
|  |  |  |  | Adjusted | 1.02 | 0.92 | 1.12 |  | 1.03 | 0.97 | 1.10 |  | 0.742 |
|  |  |  | Girls/ women | Crude | 1.02 | 0.88 | 1.18 |  | 1.01 | 0.97 | 1.06 |  | 0.962 |
|  |  |  |  | Adjusted | 1.02 | 0.88 | 1.18 |  | 1.02 | 0.97 | 1.07 |  | 0.987 |
|  |  |  | p (sex) | Crude |  |  |  | 0.962 |  |  |  | 0.924 |  |
|  |  |  |  | Adjusted |  |  |  | 0.965 |  |  |  | 0.838 |  |
|  |  | Pandemic | Boys/ men | Crude | 1.18 | 0.95 | 1.46 |  | 1.09 | 0.96 | 1.23 |  | 0.516 |
|  |  |  |  | Adjusted | 1.17 | 0.95 | 1.46 |  | 1.17 | 1.04 | 1.33 |  | 0.989 |
|  |  |  | Girls/ women | Crude | 1.20 | 0.89 | 1.61 |  | 1.08 | 1.02 | 1.15 |  | 0.521 |
|  |  |  |  | Adjusted | 1.17 | 0.87 | 1.58 |  | 1.11 | 1.05 | 1.18 |  | 0.665 |
|  |  |  | p (sex) | Crude |  |  |  | 0.943 |  |  |  | 0.958 |  |
|  |  |  |  | Adjusted |  |  |  | 0.976 |  |  |  | 0.580 |  |
|  |  | p (per) | Boys/ men | Crude |  |  |  | 0.210 |  |  |  | 0.354 |  |
|  |  |  |  | Adjusted |  |  |  | 0.208 |  |  |  | 0.356 |  |
|  |  |  | Girls/ women | Crude |  |  |  | 0.339 |  |  |  | 0.092 |  |
|  |  |  |  | Adjusted |  |  |  | 0.341 |  |  |  | 0.110 |  |
|  | Private healthcare among those with public healthcare | Pre-pandemic | Boys/ men | Crude | 1.00 | 0.91 | 1.11 |  | 0.60 | 0.56 | 0.64 |  | <0.001 |
|  |  |  |  | Adjusted | 1.02 | 0.92 | 1.13 |  | 0.69 | 0.65 | 0.73 |  | <0.001 |
|  |  |  | Girls/ women | Crude | 1.04 | 0.90 | 1.20 |  | 0.69 | 0.66 | 0.72 |  | <0.001 |
|  |  |  |  | Adjusted | 1.04 | 0.90 | 1.21 |  | 0.71 | 0.68 | 0.74 |  | <0.001 |
|  |  |  | p (sex) | Crude |  |  |  | 0.648 |  |  |  | <0.001 |  |
|  |  |  |  | Adjusted |  |  |  | 0.619 |  |  |  | <0.001 |  |
|  |  | Pandemic | Boys/ men | Crude | 0.77 | 0.62 | 0.95 |  | 0.38 | 0.34 | 0.43 |  | <0.001 |
|  |  |  |  | Adjusted | 0.77 | 0.62 | 0.96 |  | 0.47 | 0.41 | 0.53 |  | <0.001 |
|  |  |  | Girls/ women | Crude | 0.68 | 0.51 | 0.92 |  | 0.56 | 0.52 | 0.59 |  | 0.191 |
|  |  |  |  | Adjusted | 0.68 | 0.50 | 0.91 |  | 0.55 | 0.52 | 0.59 |  | 0.166 |
|  |  |  | p (sex) | Crude |  |  |  | 0.527 |  |  |  | <0.001 |  |
|  |  |  |  | Adjusted |  |  |  | 0.525 |  |  |  | <0.001 |  |
|  |  | p (per) | Boys/ men | Crude |  |  |  | 0.029 |  |  |  | <0.001 |  |
|  |  |  |  | Adjusted |  |  |  | 0.026 |  |  |  | <0.001 |  |
|  |  |  | Girls/ women | Crude |  |  |  | 0.012 |  |  |  | <0.001 |  |
|  |  |  |  | Adjusted |  |  |  | 0.012 |  |  |  | <0.001 |  |
| Primary health-care | Public healthcare among those with private healthcare | Pre-pandemic | Boys/ men | Crude | 0.97 | 0.88 | 1.08 |  | 1.03 | 0.97 | 1.09 |  | 0.389 |
|  |  |  |  | Adjusted | 0.98 | 0.88 | 1.09 |  | 1.05 | 0.99 | 1.12 |  | 0.213 |
|  |  |  | Girls/ women | Crude | 1.01 | 0.88 | 1.17 |  | 1.03 | 0.98 | 1.08 |  | 0.861 |
|  |  |  |  | Adjusted | 1.02 | 0.88 | 1.18 |  | 1.03 | 0.99 | 1.08 |  | 0.794 |
|  |  |  | p (sex) | Crude |  |  |  | 0.665 |  |  |  | 0.997 |  |
|  |  |  |  | Adjusted |  |  |  | 0.671 |  |  |  | 0.874 |  |
|  |  | Pandemic | Boys/ men | Crude | 0.99 | 0.76 | 1.29 |  | 1.13 | 0.99 | 1.28 |  | 0.393 |
|  |  |  |  | Adjusted | 0.99 | 0.76 | 1.28 |  | 1.26 | 1.11 | 1.44 |  | 0.099 |
|  |  |  | Girls/ women | Crude | 1.09 | 0.77 | 1.54 |  | 1.14 | 1.07 | 1.21 |  | 0.805 |
|  |  |  |  | Adjusted | 1.07 | 0.76 | 1.50 |  | 1.19 | 1.12 | 1.27 |  | 0.603 |
|  |  |  | p (sex) | Crude |  |  |  | 0.665 |  |  |  | 0.870 |  |
|  |  |  |  | Adjusted |  |  |  | 0.697 |  |  |  | 0.581 |  |
|  |  | p (per) | Boys/ men | Crude |  |  |  | 0.899 |  |  |  | 0.207 |  |
|  |  |  |  | Adjusted |  |  |  | 0.886 |  |  |  | 0.209 |  |
|  |  |  | Girls/ women | Crude |  |  |  | 0.697 |  |  |  | 0.008 |  |
|  |  |  |  | Adjusted |  |  |  | 0.683 |  |  |  | 0.012 |  |
|  | Private healthcare among those with public healthcare | Pre-pandemic | Boys/ men | Crude | 1.01 | 0.91 | 1.12 |  | 0.59 | 0.56 | 0.63 |  | <0.001 |
|  |  |  |  | Adjusted | 1.02 | 0.92 | 1.13 |  | 0.68 | 0.64 | 0.72 |  | <0.001 |
|  |  |  | Girls/ women | Crude | 1.09 | 0.94 | 1.26 |  | 0.69 | 0.66 | 0.73 |  | <0.001 |
|  |  |  |  | Adjusted | 1.09 | 0.94 | 1.26 |  | 0.71 | 0.68 | 0.74 |  | <0.001 |
|  |  |  | p (sex) | Crude |  |  |  | 0.398 |  |  |  | <0.001 |  |
|  |  |  |  | Adjusted |  |  |  | 0.376 |  |  |  | <0.001 |  |
|  |  | Pandemic | Boys/ men | Crude | 0.80 | 0.61 | 1.04 |  | 0.40 | 0.35 | 0.45 |  | <0.001 |
|  |  |  |  | Adjusted | 0.80 | 0.62 | 1.04 |  | 0.47 | 0.42 | 0.54 |  | 0.001 |
|  |  |  | Girls/ women | Crude | 0.74 | 0.52 | 1.04 |  | 0.58 | 0.55 | 0.62 |  | 0.191 |
|  |  |  |  | Adjusted | 0.73 | 0.52 | 1.02 |  | 0.57 | 0.53 | 0.60 |  | 0.143 |
|  |  |  | p (sex) | Crude |  |  |  | 0.710 |  |  |  | <0.001 |  |
|  |  |  |  | Adjusted |  |  |  | 0.706 |  |  |  | <0.001 |  |
|  |  | p (per) | Boys/ men | Crude |  |  |  | 0.107 |  |  |  | <0.001 |  |
|  |  |  |  | Adjusted |  |  |  | 0.107 |  |  |  | <0.001 |  |
|  |  |  | Girls/ women | Crude |  |  |  | 0.040 |  |  |  | <0.001 |  |
|  |  |  |  | Adjusted |  |  |  | 0.040 |  |  |  | <0.001 |  |
| Psychiatric health-care | Public healthcare among those with private healthcare | Pre-pandemic | Boys/ men | Crude | 8.04 | 6.92 | 9.34 |  | 7.26 | 6.67 | 7.90 |  | 0.247 |
|  |  |  |  | Adjusted | 7.74 | 6.66 | 9.00 |  | 5.01 | 4.60 | 5.47 |  | <0.001 |
|  |  |  | Girls/ women | Crude | 9.76 | 7.76 | 12.28 |  | 6.66 | 6.24 | 7.12 |  | 0.002 |
|  |  |  |  | Adjusted | 9.31 | 7.40 | 11.71 |  | 5.25 | 4.91 | 5.62 |  | <0.001 |
|  |  |  | p (sex) | Crude |  |  |  | 0.165 |  |  |  | 0.122 |  |
|  |  |  |  | Adjusted |  |  |  | 0.139 |  |  |  | 0.257 |  |
|  |  | Pandemic | Boys/ men | Crude | 4.61 | 3.15 | 6.74 |  | 9.30 | 7.51 | 11.51 |  | 0.002 |
|  |  |  |  | Adjusted | 4.35 | 2.97 | 6.37 |  | 5.78 | 4.65 | 7.17 |  | 0.335 |
|  |  |  | Girls/ women | Crude | 2.81 | 1.50 | 5.28 |  | 8.86 | 7.92 | 9.90 |  | <0.001 |
|  |  |  |  | Adjusted | 2.24 | 1.19 | 4.20 |  | 7.10 | 6.35 | 7.95 |  | 0.006 |
|  |  |  | p (sex) | Crude |  |  |  | 0.189 |  |  |  | 0.690 |  |
|  |  |  |  | Adjusted |  |  |  | 0.121 |  |  |  | 0.087 |  |
|  |  | p (per) | Boys/ men | Crude |  |  |  | 0.008 |  |  |  | 0.034 |  |
|  |  |  |  | Adjusted |  |  |  | 0.005 |  |  |  | 0.037 |  |
|  |  |  | Girls/ women | Crude |  |  |  | <0.001 |  |  |  | <0.001 |  |
|  |  |  |  | Adjusted |  |  |  | <0.001 |  |  |  | <0.001 |  |
|  | Private healthcare among those with public healthcare | Pre-pandemic | Boys/ men | Crude | 1.08 | 0.93 | 1.26 |  | 0.72 | 0.66 | 0.78 |  | <0.001 |
|  |  |  |  | Adjusted | 1.03 | 0.89 | 1.20 |  | 0.78 | 0.72 | 0.85 |  | <0.001 |
|  |  |  | Girls/ women | Crude | 0.98 | 0.78 | 1.23 |  | 0.80 | 0.75 | 0.85 |  | 0.092 |
|  |  |  |  | Adjusted | 0.95 | 0.75 | 1.20 |  | 0.84 | 0.78 | 0.89 |  | 0.178 |
|  |  |  | p (sex) | Crude |  |  |  | 0.469 |  |  |  | 0.048 |  |
|  |  |  |  | Adjusted |  |  |  | 0.438 |  |  |  | 0.061 |  |
|  |  | Pandemic | Boys/ men | Crude | 0.97 | 0.66 | 1.41 |  | 0.52 | 0.42 | 0.64 |  | 0.005 |
|  |  |  |  | Adjusted | 0.97 | 0.66 | 1.42 |  | 0.60 | 0.49 | 0.75 |  | 0.036 |
|  |  |  | Girls/ women | Crude | 0.52 | 0.28 | 0.98 |  | 0.70 | 0.63 | 0.79 |  | 0.358 |
|  |  |  |  | Adjusted | 0.52 | 0.28 | 0.97 |  | 0.77 | 0.69 | 0.86 |  | 0.234 |
|  |  |  | p (sex) | Crude |  |  |  | 0.100 |  |  |  | 0.012 |  |
|  |  |  |  | Adjusted |  |  |  | 0.095 |  |  |  | 0.014 |  |
|  |  | p (per) | Boys/ men | Crude |  |  |  | 0.581 |  |  |  | 0.006 |  |
|  |  |  |  | Adjusted |  |  |  | 0.767 |  |  |  | 0.004 |  |
|  |  |  | Girls/ women | Crude |  |  |  | 0.065 |  |  |  | 0.059 |  |
|  |  |  |  | Adjusted |  |  |  | 0.071 |  |  |  | 0.046 |  |
| Somatic health-care | Public healthcare among those with private healthcare | Pre-pandemic | Boys/ men | Crude | 1.11 | 1.00 | 1.24 |  | 0.95 | 0.88 | 1.01 |  | 0.010 |
|  |  |  |  | Adjusted | 1.12 | 1.01 | 1.24 |  | 1.04 | 0.97 | 1.11 |  | 0.291 |
|  |  |  | Girls/ women | Crude | 1.17 | 1.01 | 1.35 |  | 1.02 | 0.97 | 1.07 |  | 0.081 |
|  |  |  |  | Adjusted | 1.17 | 1.01 | 1.36 |  | 1.05 | 1.00 | 1.10 |  | 0.187 |
|  |  |  | p (sex) | Crude |  |  |  | 0.606 |  |  |  | 0.089 |  |
|  |  |  |  | Adjusted |  |  |  | 0.614 |  |  |  | 0.290 |  |
|  |  | Pandemic | Boys/ men | Crude | 1.33 | 1.04 | 1.71 |  | 1.14 | 0.99 | 1.31 |  | 0.281 |
|  |  |  |  | Adjusted | 1.32 | 1.03 | 1.71 |  | 1.39 | 1.21 | 1.59 |  | 0.750 |
|  |  |  | Girls/ women | Crude | 1.27 | 0.89 | 1.82 |  | 1.08 | 1.01 | 1.17 |  | 0.397 |
|  |  |  |  | Adjusted | 1.24 | 0.86 | 1.77 |  | 1.16 | 1.08 | 1.25 |  | 0.672 |
|  |  |  | p (sex) | Crude |  |  |  | 0.832 |  |  |  | 0.543 |  |
|  |  |  |  | Adjusted |  |  |  | 0.798 |  |  |  | 0.089 |  |
|  |  | p (per) | Boys/ men | Crude |  |  |  | 0.197 |  |  |  | 0.019 |  |
|  |  |  |  | Adjusted |  |  |  | 0.187 |  |  |  | 0.020 |  |
|  |  |  | Girls/ women | Crude |  |  |  | 0.670 |  |  |  | 0.150 |  |
|  |  |  |  | Adjusted |  |  |  | 0.642 |  |  |  | 0.240 |  |
|  | Private healthcare among those with public healthcare | Pre-pandemic | Boys/ men | Crude | 0.96 | 0.86 | 1.06 |  | 0.59 | 0.55 | 0.63 |  | <0.001 |
|  |  |  |  | Adjusted | 0.98 | 0.88 | 1.08 |  | 0.66 | 0.62 | 0.71 |  | <0.001 |
|  |  |  | Girls/ women | Crude | 0.96 | 0.83 | 1.11 |  | 0.70 | 0.67 | 0.74 |  | <0.001 |
|  |  |  |  | Adjusted | 0.96 | 0.83 | 1.12 |  | 0.71 | 0.68 | 0.75 |  | <0.001 |
|  |  |  | p (sex) | Crude |  |  |  | 0.965 |  |  |  | <0.001 |  |
|  |  |  |  | Adjusted |  |  |  | 0.930 |  |  |  | <0.001 |  |
|  |  | Pandemic | Boys/ men | Crude | 0.71 | 0.55 | 0.91 |  | 0.45 | 0.39 | 0.51 |  | 0.002 |
|  |  |  |  | Adjusted | 0.71 | 0.55 | 0.92 |  | 0.54 | 0.47 | 0.62 |  | 0.059 |
|  |  |  | Girls/ women | Crude | 0.54 | 0.38 | 0.77 |  | 0.58 | 0.54 | 0.62 |  | 0.701 |
|  |  |  |  | Adjusted | 0.53 | 0.37 | 0.77 |  | 0.57 | 0.53 | 0.61 |  | 0.788 |
|  |  |  | p (sex) | Crude |  |  |  | 0.216 |  |  |  | 0.001 |  |
|  |  |  |  | Adjusted |  |  |  | 0.214 |  |  |  | 0.001 |  |
|  |  | p (per) | Boys/ men | Crude |  |  |  | 0.032 |  |  |  | <0.001 |  |
|  |  |  |  | Adjusted |  |  |  | 0.028 |  |  |  | 0.001 |  |
|  |  |  | Girls/ women | Crude |  |  |  | 0.003 |  |  |  | <0.001 |  |
|  |  |  |  | Adjusted |  |  |  | 0.003 |  |  |  | <0.001 |  |
